# The role of MRI in radiotherapy planning: a narrative review “from head to toe”

**DOI:** 10.1186/s13244-024-01799-1

**Published:** 2024-10-23

**Authors:** Simona De Pietro, Giulia Di Martino, Mara Caroprese, Angela Barillaro, Sirio Cocozza, Roberto Pacelli, Renato Cuocolo, Lorenzo Ugga, Francesco Briganti, Arturo Brunetti, Manuel Conson, Andrea Elefante

**Affiliations:** 1 Department of Advanced Biomedical Sciences, University of Naples “Federico II”, Naples, Italy; 2 Department of Medicine, Surgery and Dentistry, University of Salerno, Baronissi, Italy

**Keywords:** Radiotherapy planning, Magnetic resonance imaging, Radiation oncology

## Abstract

**Abstract:**

Over the last few years, radiation therapy (RT) techniques have evolved very rapidly, with the aim of conforming high-dose volume tightly to a target. Although to date CT is still considered the imaging modality for target delineation, it has some known limited capabilities in properly identifying pathologic processes occurring, for instance, in soft tissues. This limitation, along with other advantages such as dose reduction, can be overcome using magnetic resonance imaging (MRI), which is increasingly being recognized as a useful tool in RT clinical practice. This review has a two-fold aim of providing a basic introduction to the physics of MRI in a narrative way and illustrating the current knowledge on its application “from head to toe” (i.e., different body sites), in order to highlight the numerous advantages in using MRI to ensure the best therapeutic response. We provided a basic introduction for residents and non-radiologist on the physics of MR and reported evidence of the advantages and future improvements of MRI in planning a tailored radiotherapy treatment “from head to toe”.

**Critical relevance statement:**

This review aims to help understand how MRI has become indispensable, not only to better characterize and evaluate lesions, but also to predict the evolution of the disease and, consequently, to ensure the best therapeutic response.

**Key Points:**

- MRI is increasingly gaining interest and applications in RT planning.
- MRI provides high soft tissue contrast resolution and accurate delineation of the target volume.
- MRI will increasingly become indispensable for characterizing and evaluating lesions, and to predict the evolution of disease.

**Graphical Abstract:**

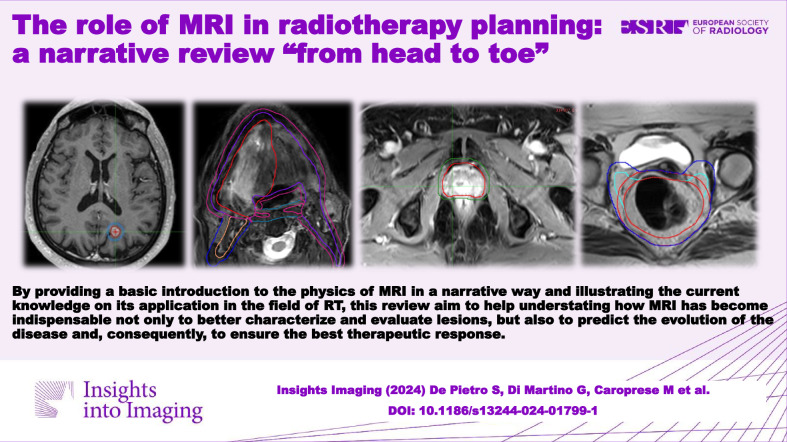

## Introduction

Radiation therapy (RT) techniques have evolved over the last decades, along with the capacity to conform high dose volume tightly to the target. Technical improvements, mostly related to the development of hardware and software tools, have consisted of advances in imaging and radiation delivery technology at each stage of the multistep process of RT (i.e., from plan preparation to patient treatment), resulting in an improved toxicity profile. Currently, most radiotherapy treatments are delivered through intensity-modulated radiation therapy (IMRT). This advanced technique involves the delivery of a non-uniform dose to the treatment volume, which could ideally be divided into subvolumes according to different morpho-functional characteristics. It goes without saying that these features can be identified by multimodal imaging. Given this background, it is easy to understand how the use of different imaging modalities for planning preparation represents a mandatory requirement for modern RT.

Computed tomography (CT) was introduced in RT more than 30 years ago, and it was the trigger for achieving the dose conformation objective. It still represents, to date, the routine modality for RT planning preparation, providing density maps of the treated area which represent the basis for the operation of the dose calculation algorithms in external beam RT. Furthermore, CT is the basic imaging modality for target delineation due to its high spatial resolution and reproducibility, especially given that this technique does not suffer from geometric distortions. Nevertheless, it should be highlighted that, in many clinical settings, CT-based delineation might not be sufficient for an appropriate definition of the target. Indeed, while lesion demarcation is relatively accurate for tumors adjacent to air, bone, or fat, the identification of normal or pathologic processes occurring in soft tissues is, to some extent, relatively limited. This limitation can be overcome by the use of magnetic resonance imaging (MRI), a non-invasive technique that, using high-field magnets and radiofrequency (RF), allows the acquisition of images of different human body sites. Its main advantages, compared to CT, lie in the greater soft tissue contrast resolution (even without contrast agent administration), its lack of ionizing radiation, the possibility of tailoring the acquisition protocol to the region of interest, and of employing advanced sequences for functional imaging.

This review has the aim of illustrating the current knowledge about the application of MRI in RT planning. It consists of two main sections: the first one introduces the basics of the physical phenomena of MRI (written in a narrative way, from physicians to physicians, therefore deliberately not including equations with the aim of increasing its readability for all radiology, oncology, and radiation oncology residents, as well as non-radiologists physicians), and a second one indicating, for each main body area, the advantages of applying MRI to RT planning, including the benefits of MR-guided RT (MRgRT).

## MRI basic principles

To obtain a signal from MRI scans, it is firstly necessary to expose the subject to a strong and constant magnetic field (B0) with a strength usually of 1.5 Tesla (T) or 3 T.

Protons contained in the hydrogen nuclei are constantly spinning, generating small electrical currents, and consequently, small magnetic fields, known as magnetic moments. In the absence of an external magnetic field, the orientation of these magnetic moments, known as nuclear spin, is isotropic. When an external magnetic field is applied, these spins modify their orientation, which could be parallel or antiparallel to the orientation of the B0. In addition to this energy split, the application of a B0 will also produce a precession of the magnetic moments around the main magnetic field dependent on the strength of the magnetic field B0 and from a constant specific to the particular nuclear species named gyromagnetic ratio.

**Fig. 1:**
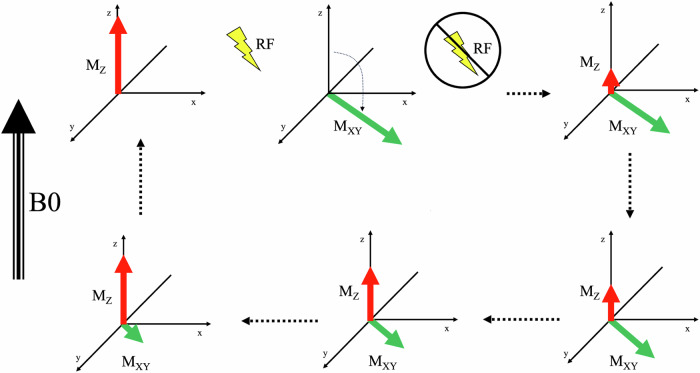
Schematic representation of the two main different types of relaxation, namely the longitudinal (or T1) and the transverse (or T2) relaxation, which correspond to the increase to original values of *M*_Z_ (red arrow) and the disappearance of the *M*_*XY*_ (green arrow), respectively, after the removal of the radiofrequency pulse (yellow bolt)

**Fig. 2:**
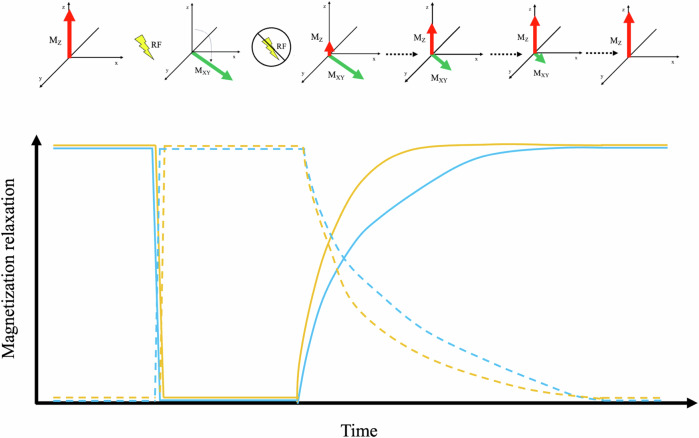
Schematic representation of the relation between *M* relaxation and time, showing the increase in longitudinal relaxation (full lines) occurring along with the decrease in the transverse one (dashed lines) for two different molecules (e.g., water and fat in blue and yellow, respectively)

The averaged sum of all the individual spins in a specific sample is called net magnetization (*M*), which behaves like a vector aligned with B0 at equilibrium and that can be resolved in two main components, namely the longitudinal (*M*_Z_) and the transversal (*M*_*XY*_) magnetization. When an RF pulse is applied, protons fall out of the alignment with B0, displacing *M* and making it precess. When the RF pulse is removed, the protons return to equilibrium, following a process called *M* relaxation. There are two main different types of relaxation, namely the longitudinal (or the T1 time constant) and the transverse (or the T2 time constant) relaxation, which correspond to the increase to original values of *M*_Z_ and the disappearance of the *M*_*XY*_, respectively (Figs. 1 and 2).

In particular, longitudinal (T1) relaxation is determined by the restoration of the net *M* due to the exchange of energy between protons and their surroundings in returning to a lower energy status. This exchange depends on the tumbling rate and dimensions of the examined molecule: free water has a small molecular size and shows a very high tumbling rate, and it is therefore characterized by a long T1 relaxation time (≈ 4000 ms), while when free water is bound in more complex structures, such as proteins or lipids, the tumbling rate decreases leading to shorter T1 relaxation time (e.g., ≈ 250 ms for fat).

On the other hand, transversal (T2) relaxation time depends on the time protons need to fall out of phase on the *XY* plane, leading to a decrease and subsequent disappearance of *M*_*XY*_, which can occur independently from energy exchange. This loss of phase is mainly caused by local inhomogeneity due to interactions between the spin of adjacent protons, with subsequent loss of phase coherence. The spin–spin interaction is influenced by the characteristics of the examined structure. In tissues with complex structures, more spin–spin interactions occur leading to shorter T2 relaxation time compared to free water, in which proton interactions are less favored (≈ 50 ms for muscles vs ≈ 2000 ms for water). Nevertheless, inhomogeneities produced by spin–spin interaction are not the only ones occurring, since global inhomogeneities within B0 are also present. The interaction between T2 relaxation and such inhomogeneity goes under the name of T2* relaxation time, and it is also referred to as the “observed” T2 relaxation, since it reflects both the true protons interaction along the magnetic field inhomogeneity.

Other basic concepts that need to be introduced are the repetition time (TR) and the echo time (TE). The first one is the time intercurring between the two successive RF pulses, while the second represents the time that passes between the application of an RF and the detection of the peak of a signal. These two parameters directly influence T1 and T2 relaxation times, and the contrast of the images that are subsequently generated. In particular, an acquisition with short TR and short TE produces T1-weighted (T1w) images, because the short TR will not allow for a complete recovery of *M*_Z_ (therefore maximizing the contribution of T1), while the short TE limits the T2 effect on the image. On the other hand, sequences with a long TR and long TE will produce T2-weighted (T2w) images, because the long TR will limit the T1 effect, while a long TE will then provide a more pronounced T2 effect. In clinical practice, T1w images are usually acquired because they produce good anatomical images, while the sensitivity in the detection of pathological processes is relatively low. On the other hand, T2w images show a high sensitivity in the detection of pathological processes, due to the higher sensitivity to water changes (i.e., edema, gliosis, etc.) often occurring in pathological processes. Finally, it is also important to remember that TR directly influences the length of an acquisition, while TE has no direct effect on the duration of the sequence.

Given this background, it is possible to say that in clinical practice there are two main sequences that are routinely acquired: spin echo (SE) and gradient echo (GrE) sequences. SE sequences, generated by administering a pair of successive RFs (usually at 90° and 180°, respectively), are slow sequences as they require the application of two distinct RF pulses, but are relatively resistant to some types of artifacts (e.g., field inhomogeneities or susceptibility). On the other hand, GrE sequences are produced by using a single RF pulse, along with a gradient reversal rather than the 180° RF pulse to refocus the spins. The use of gradients leads to shorter TRs and TEs compared to SE sequences, in exchange for high signal loss in the presence of magnetic susceptibility effects. Gradients, which are loops of conductive wire on a cylindrical shell, are major components of the MR scanner. These are in number of three (*x*, *y*, and *z*) each connected to an independent power amplifier, and are used to create a secondary magnetic field (through the application of a current) called a “gradient field”. The distortion induced by the application of this second field is predictable, and therefore used by the operator to allow spatial encoding of the MR signal.

## Brain

In the field of neuro-oncology, MRI is widely recognized as the imaging standard of reference due to its superior soft tissue contrast resolution.

Although a large degree of variability is present, on conventional MRI sequences brain lesions usually present with low to intermediate signal intensity on T1w, coupled to a mild to moderate hyperintensity on T2w sequences, with the administration of gadolinium-based contrast agents that help to visualize the blood–brain barrier breakdown and better evaluate the solid portion of a lesion.

**Fig. 3:**
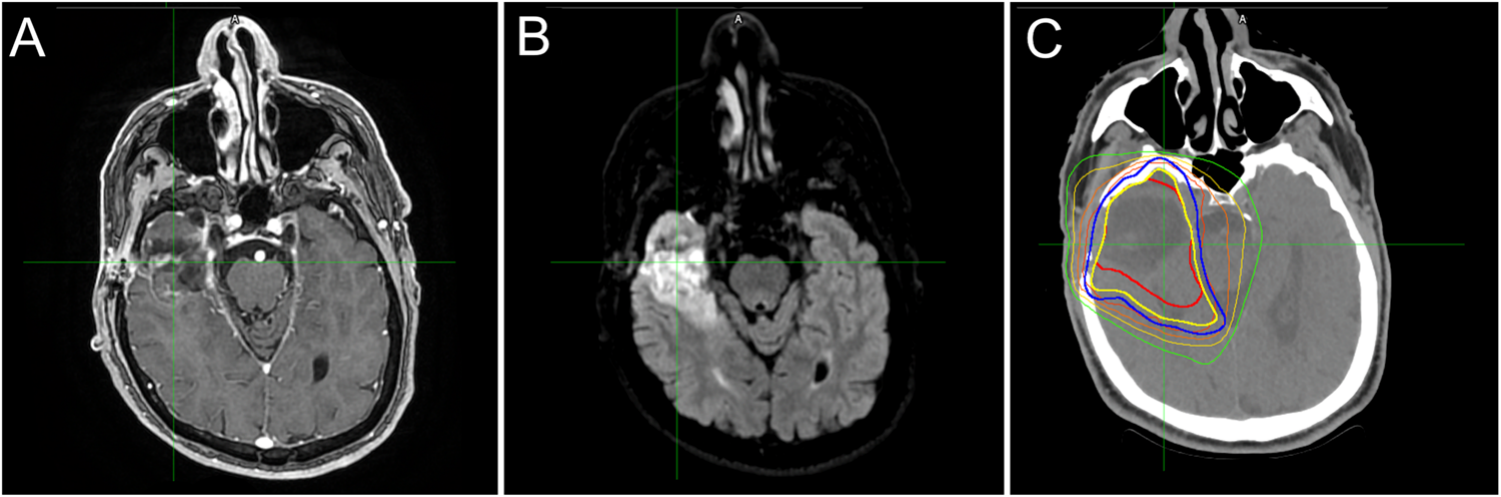
Contrast-enhanced T1w (**A**) and FLAIR-weighted (**B**) images, obtained after surgery of a lesion consistent with a glioblastoma, demonstrate the surgical cavity with peripheral signal abnormality, which is included in the distribution of doses of the centering CT (**C**)

**Fig. 4:**
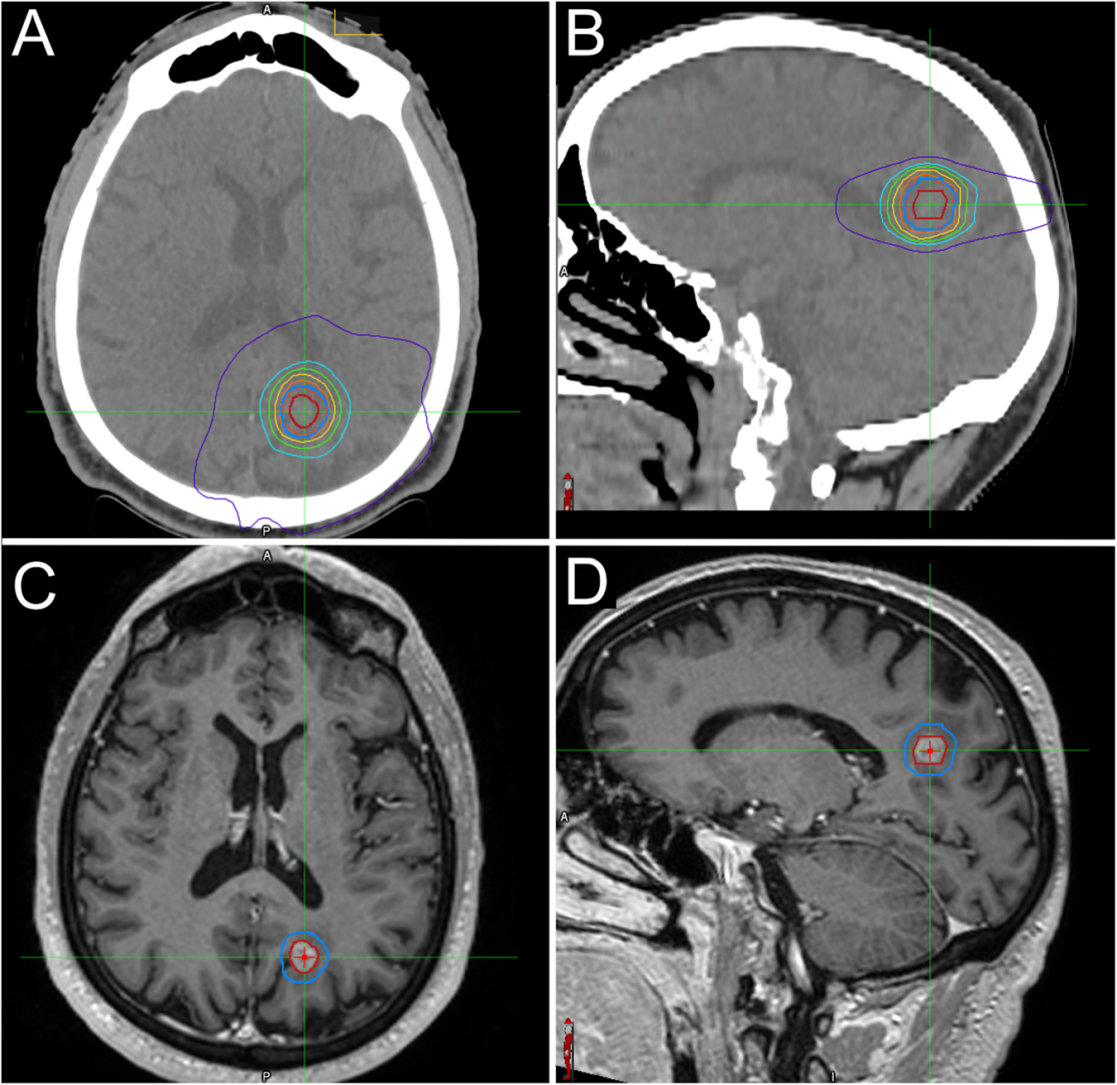
Axial (**A**) and sagittal (**B**) reformats of the centering CT with the superimposed distribution of doses, and axial (**C**) and sagittal (**D**) reformats of a volumetric contrast-enhanced T1w sequence with GTV (red line) and PTV (blue line) in a patient with lung cancer brain metastasis

For brain RT planning, MRI sequences preferentially used are post-gadolinium 3D isotropic sequences, as they reduce geometric distortions allowing accurate estimation of gross tumor volume, both reducing partial volume effects (Fig. 3) and also allowing to detection of small secondary lesions (Fig. 4).

In particular, it has been demonstrated that 3D SE T1w sequences are superior to their GrE equivalent in delineating the target volume of intracranial radiotherapy [1]. Indeed, despite their lower contrast between gray and white matter, SE sequences allow for the delineation of smaller intracranial metastases more clearly, also reducing metal artifacts in patients with shunts and/or surgical clips [2].

With reference to the “functional” and more advanced techniques, the first that needs to be introduced, given its prominent role in clinical practice, is perfusion-weighted imaging (PWI). This technique allows for the evaluation of different perfusion-related parameters both in the lesion and the surrounding brain parenchyma, aiding not only in the distinction between tumor and edema, but also contributing to grade assessment. Currently, the commonly used techniques of perfusion imaging are dynamic susceptibility contrast (DSC) and, to a lesser extent, dynamic contrast enhancement (DCE) sequences. The DSC-PWI relies on the susceptibility-induced signal loss on T2*w sequences following the fast administration of a bolus of gadolinium-based contrast agent [3]. Although several metrics can be obtained from this technique, the most robust and clinically validated parameter is the relative cerebral blood volume, which is known to be very useful not only for the diagnosis, but also for follow-up examinations, being the only sequence able to differentiate between true progression and post-RT pseudo-progression. Regarding the DCE-PWI, it suffers from a lack of standardization in terms of acquisition parameters and processing procedures, making it not widely used [4].

Another important role among advanced MRI techniques used for RT planning is played by diffusion-weighted imaging (DWI), and in particular diffusion tensor imaging, an accurate method not only to assess tissue microstructure integrity, but also to reconstruct white matter bundles via tractography analysis, to preserve them as much as possible when planning target volume (PTV) coverage without treatment efficacy reduction [5].

Finally, other advanced MRI techniques, such as relaxometry, quantitative susceptibility mapping, or magnetic resonance fingerprinting, although all very promising, are mostly acquired to date in research settings [6–8], as a full implementation in clinical practice must require the build-up of a more wide and consistent scientific literature.

## Head and neck

The introduction of higher-dose RT techniques (e.g., intensity-modulated radiotherapy) has led to a growing interest in the use of MRI for accurate lesion delineation for head and neck tumors. This has been reflected in clinical practice by the recent introduction of dedicated MRI-RT equipment for head and neck cancer. A further evolution of this approach is represented by integrated MR-linear accelerator systems. In the head and neck region, functional images obtained from PWI and DWI are of particular interest, providing accurate characterization and demarcation of both the primary lesion and the nodal metastases [9]. Nevertheless, these techniques rely on sequences (namely, echo-planar sequences) that are prone to artifacts in this anatomical region, mainly due to the relatively high presence of air and bone.

In current practice, delineation of the target and organ at risk is mostly conducted on axial CT images. However, due to the relatively poor soft tissue contrast inherent in CT images, target delineation by CT presents great difficulties. Compared to CT, MRI provides better differentiation of soft tissues such as salivary glands and surgical flaps [10].

**Fig. 5:**
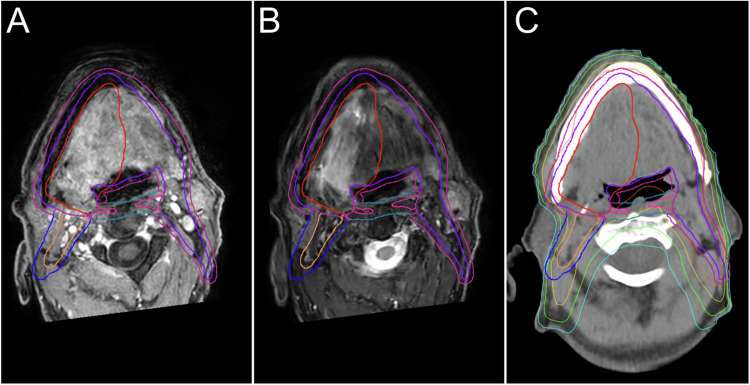
Contrast-enhanced fat-saturated T1w (**A**), STIR-weighted images (**B**), and related centering CT (**C**) with the superimposed distribution of doses, GTV (red line), and PTV (blue line) in a patient affected by oral floor squamous cell carcinoma

For anatomical delineation, T2w and contrast-enhanced T1w sequences are routinely used for RT planning. In addition to anatomical MRI, which alone does not result in a marked improvement in PTV delineation compared with CT, functional MRI techniques appear to provide numerous benefits in the clinical practice of radiation oncology. In the head and neck areas, DWI plays a key role in differentiating pathological tissue from healthy tissue (Fig. 5). It provides information on histopathology, differential diagnosis, and assessment of response to treatment [11]. To be specific, the apparent diffusion coefficient (ADC) map allows to differentiate between the tumor and peritumoral inflammatory changes, the latter not showing diffusion restriction. DWI has been useful also in identifying metastatic lymph nodes, particularly small lymph nodes, and may play an essential role in determining the extent of the RT field [12]. In addition, DWI is necessary for the evaluation of healthy tissues, such as for the study of salivary glands and the assessment of xerostomia, which is a common side effect of chemotherapy treatment. Recent studies have shown that diffusion sequences allow prediction of response to radiotherapy treatment in patients with head and neck cancers by assessing changes in ADC values, which appear to be significantly different across responder categories [13]. In particular, it seems that RT-induced reduction of tumor cells and necrosis leads to an increase in tumor ADC, and complete responders show a significant increase in ADC during/after treatment compared with nonresponders. DWI also plays an important role after radiotherapy treatment as it can help detect recurrences. It has been widely demonstrated in the literature that the ADC of residual squamous cell carcinoma is significantly lower than that of a post-treatment benign mass [14, 15].

Patients with head and neck cancers undergo numerous anatomical changes during the course of RT. This could lead to insufficient coverage by the target dose. Interfractional changes can be accounted for by applying safety margins to the target volume. However, this causes an increase in the dose allocated to normal tissues on which numerous adverse effects depend [16]. This could be solved through the employment of MRgRT, which is based on the use of an MR Linear Accelerator (MR Linac) and integrates an MRI unit with an RT unit. This allows real-time imaging of target volumes and organs at risk before and during treatment delivery, helping in assessing motion management and providing online adaptive therapy [17, 18].

## Breast

Although the diagnostic role of MRI in the preoperative management of patients with breast carcinoma is well established, the use of MRI-guided linear accelerators (LINACs) to treat patients with breast cancer does not represent the standard in clinical practice. Accelerated partial breast irradiation refers to a wide range of irradiation modalities, whose goal is to treat the lumpectomy cavity (LC) considering a well-defined margin in order to target even microscopic disease [19]. This technique allows to delivery of radiation to a limited area of the body for a shorter period than conventional whole-breast irradiation.

**Fig. 6:**
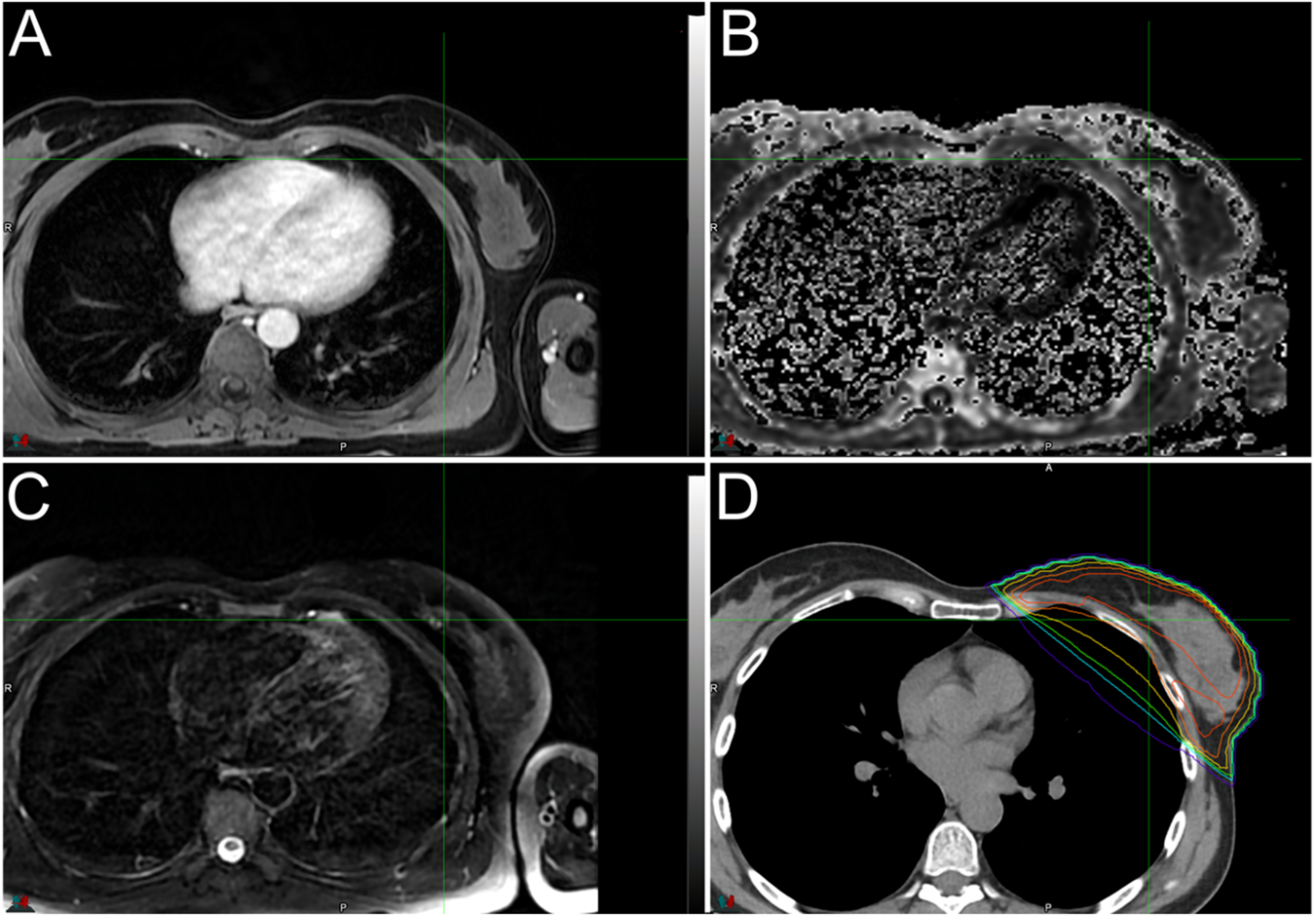
Contrast-enhanced fat-saturated T1w image (**A**), exponential ADC map (**B**), fat-suppressed T2w sequence (**C**), and related centering CT (**D**) with superimposed distribution of doses in a patient with breast cancer

Imaging has a primary role in ensuring satisfactory results in an attempt to spare biologically healthy tissue areas adjacent to the disease. Potential advantages of MRI-guided radiotherapy include ameliorated visualization of the surgical cavity after lumpectomy and improved contouring of the target volume in terms of interobserver concordance, reproducibility, and anatomical accuracy (Fig. 6). The main differences between MRI and CT in the delineation of the LC volume in the supine radiotherapy treatment position have been investigated in the literature; it has been shown that both MRI and CT succeeded in providing optimal visibility of the LC in most cases. However, while the LC is well-defined by both CT and MRI, the latter technique better depicts the interface of the LC seroma with the unaffected tissues, as well as any axillary seroma interfaces. This leads to increased confidence of radiologists and radiation oncologists in establishing a suitable treatment plan for patients [20]. In addition, as already mentioned, imaging is a useful tool for the radiotherapist in safeguarding the organs at risk adjacent to the area to be irradiated. This ambitious goal could be achieved by going for a reduction of PTV margins to about 3 mm from the conventional limit of 10 mm. Comparing the two main conventional diagnostic techniques (MRI and CT) used in planning the region of pre-therapeutic interest, it was shown that both methods enabled radiation therapists to hit the intended target when the PTV margin was set at 3 mm without significant differences between MRI and CT. However, MRI with PTV 3 mm proved significantly superior to CT PTV 10 mm in determining better sparing of the ipsilateral breast and chest wall mainly because of the difference in PTV margins [21]. One of the limitations associated with using MRI as the main imaging technique for radiotherapy treatment planning is the lack of standardization of the procedure, starting with patient positioning. For example, in diagnostics, the patient is placed in the prone position to minimize breathing artifacts, whereas the current standard of breast radiotherapy prefers the supine position to maximize available treatment angles and allow visual confirmation of treatment fields [22]. Indeed, recent acquisitions in the literature show that the prone position may even enhance the effects of the accelerated partial breast irradiation because it ensures that the LC is moved away from the heart and lung and results in a significant reduction in the radiation dose targeting these vital organs [23–25]. There is a need for the development of a standardized MRI imaging protocol usable by operators to optimally define PTV. This protocol is based on positioning the patient in the prone position with a dedicated coil exploiting customized pulse trains to emphasize contrastographic features in T1w and T2w sequences of the lumpectomy cavities and to obtain high contrastographic and spatial resolution images. Moreover, MRI can be used also to obtain useful information to predict future disease behavior. For example, MRI has been shown to perform particularly well in measuring background parenchymal enhancement associated with an increased risk of developing breast cancer [26]. Such biomarkers will add valuable information about disease progression and, therefore, may represent a further tool to adapt or modify the treatment plan.

## Prostate

**Fig. 7:**
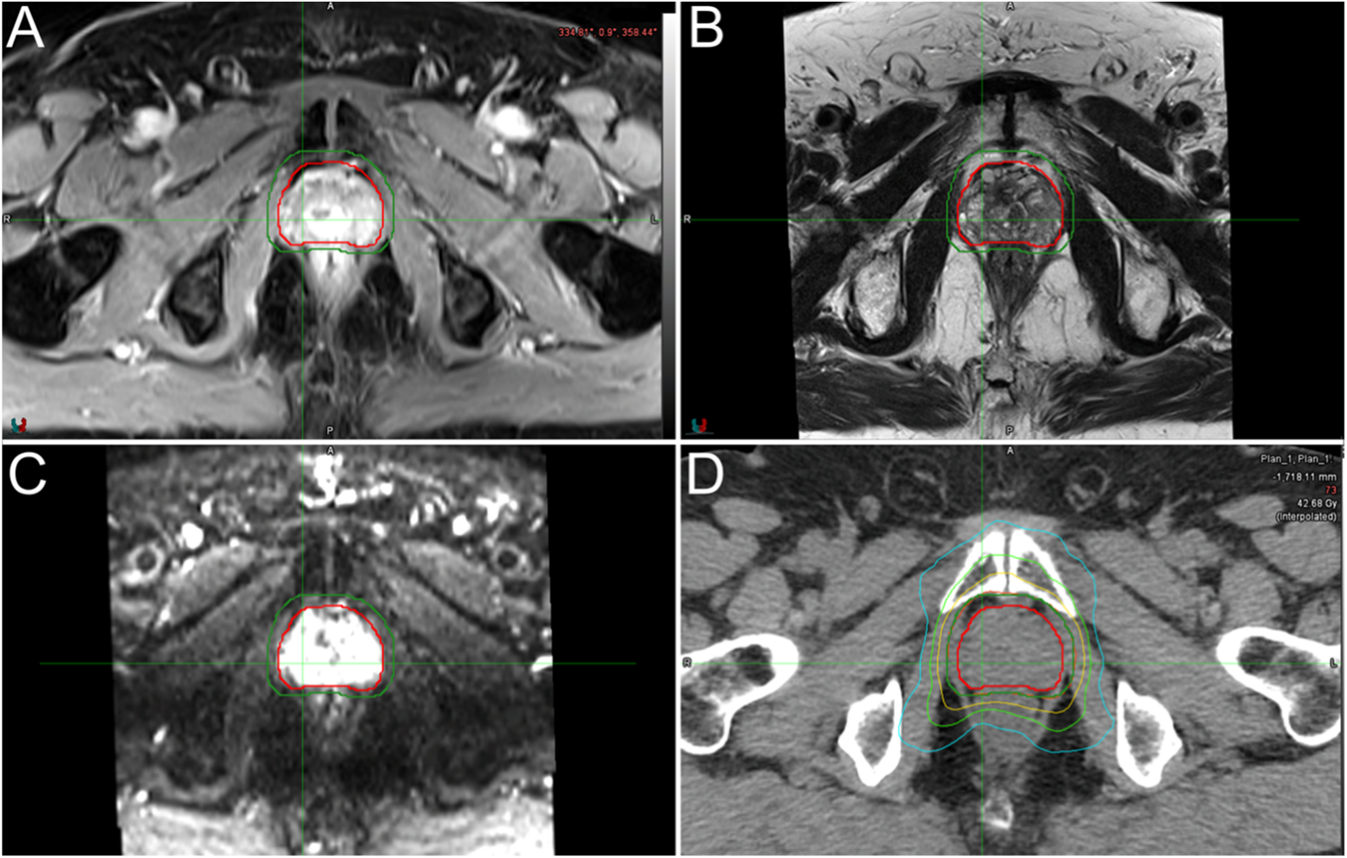
Contrast-enhanced fat-saturated T1w (**A**), T2w (**B**) images, b1000 DWI (**C**), and related centering CT (**D**) with superimposed GTV (red line), PTV (green line) and distribution of doses (**D**) in a patient with prostate cancer

MRI is the imaging technique used to assess the presence and evaluate the extension of neoplastic diseases of the prostate, as demonstrated by the well-established standardization of both image acquisition protocol and interpretation (PI-RADS). In particular, the standard multiparametric MRI protocol for prostate cancer imaging is constituted by T2w images, DWI with corresponding ADC maps, and DCE (Fig. 7). However, the latest version of the PI-RADS guidelines allow for the use of biparametric protocols (i.e., including T2w and DWI/ADC images alone) for some indications [27, 28]. In particular, for lesions localized in the transition zone, the T2w sequence is the most relevant, while DWI and ADC maps have proven to be more useful when evaluating peripheral zone cancer [29]. Pertaining to the implementation of MRI in the RT planning workflow, similar to what is reported for other anatomic regions, the main issue lies in the differences between diagnostic MRI scanners and RT simulators, especially in terms of patient positioning. As a possible solution, MRI-RT systems (or MRI simulators) have become available, with adaptations allowing for more reproducible patient positioning.

In current clinical practice, prostate cancer represents one of the areas in which the implementation of an RT MRI-only workflow has made the most progress. It is widely recognized that stereotactic body radiotherapy is a viable alternative for the treatment of prostate cancer, given its possibility to deliver large doses of radiation per day with extreme precision for a total of five or fewer treatment sessions. Although generally well tolerated, RT can result in both acute and late toxicity to the genital-urinary and intestinal tracts. In this light, LINACs could offer several advantages. For example, this technique allows for direct monitoring of prostate motion rather than using standard markers that are proxies for prostate motion and require invasive procedures to be placed [30, 31]. In addition, MRI provides increased soft tissue contrast, which also goes a long way toward improving the accuracy of alignment prior to treatment delivery. The prostate, surrounding tissues, and the interfaces between them are better demarcated in MRI than in CT [32]. Therefore, residual errors caused by fusing diagnostic MRI images to planning CT scans to delineate contours can be minimized by directly using MRI for contouring. These advantages associated with using MRI as a guide for treatment planning can result in an important reduction in the PTV margins used to ensure adequate dosing. High-dose regions of the PTV often overlap with parts of the bladder, rectum, and other nearby structures and contribute to treatment-related toxicity [33, 34]. It has been shown, comparing CT-guidance and MRI-guidance, that the latter results in a significant reduction in both the toxic effects and symptom burden experienced by patients in the acute phase immediately following treatment [35].

Currently, moderate hypofractionation is the standard in radiotherapy for the treatment of localized prostate cancer, providing no less oncologic control of the disease than normal fractionation and reduction of therapy-related toxicity, while extreme hypofractionation (e.g., 4–7 fractions) seems to be the next step in further reducing the overall treatment time, showing, compared to standard fractionation, comparable outcomes with comparable or only slightly increased toxicity. This is possible mainly because prostate cancer is characterized by a low α/β value and, consequently, a large radiation dose per fraction (the principle on which hypofractionation is based) can reasonably result in increased treatment efficacy [36, 37]. Ultra-fractionation, together with the use of MRI as linear accelerator guidance, was shown to be particularly effective in increasing treatment efficacy and reducing toxicity at the same time [38].

## Rectum

For local staging of rectal cancer, MRI is again considered the imaging standard of reference. For this reason, it is already routinely embraced for tumor delineation in RT planning.

The protocol adopted nowadays involves the acquisition of TSE-T2w sequences in the axial, coronal, and sagittal planes for evaluation of the T-category of the tumor. Furthermore, an axial sequence perpendicular to the tumor and a coronal sequence parallel to the tumor must be performed to avoid partial volume effects, and all these images must be acquired at a maximum slice thickness of 3 mm [39]. When the tumor is near the anal sphincter complex, an additional high-resolution oblique coronal TSE-T2W parallel to the anal canal should be performed to assess the relationship of the tumor to the sphincter complex [40].

Rectal cancer usually shows increased enhancement after contrast administration, along with restricted water diffusion due to high cellularity. Nevertheless, MRI also allows for an accurate depiction of the mesorectal fascia and its surrounding fat, assessing lesion infiltration of these structures, without the need for contrast agent administration [41].

**Fig. 8:**
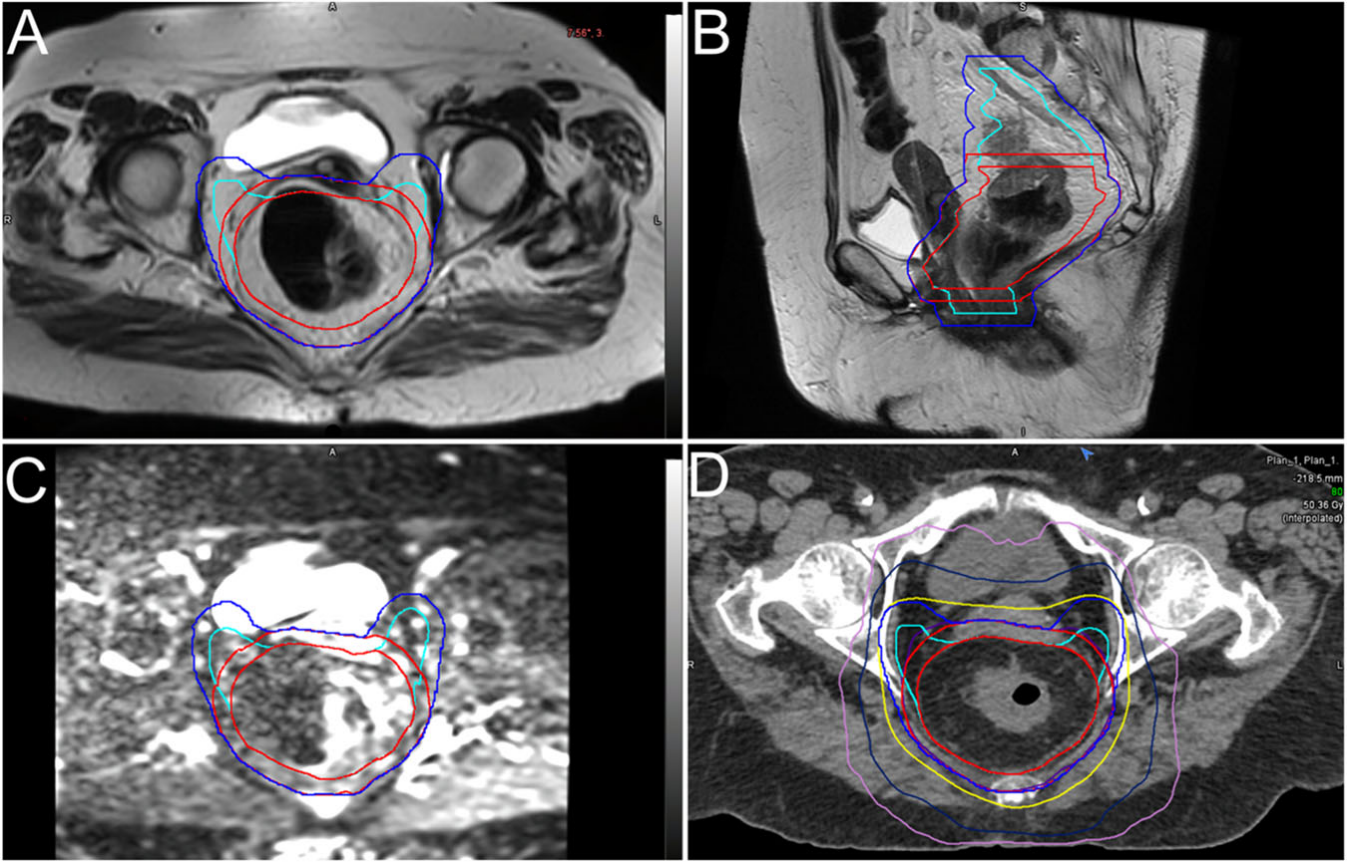
Axial T2- (**A**), sagittal T2w (**B**) images, b1000 DWI (**C**), and related centering CT (**D**) with superimposed GTV (red line), PTV (blue line) and distribution of doses in a patient with rectal cancer

The integration of different advanced MRI techniques (e.g., DWI or PWI) is also useful in locoregional nodal staging, as the correct identification of positive lymph nodes can be challenging in morphological imaging [42]. For example, it has been shown that the addition of DWI to the T2w sequences can increase sensitivity from 82–84% to 93–95% in contouring studies [43] (Fig. 8). Furthermore, considering the minimal amount of time required to include DWI in the MRI protocol of these patients, it is now considered necessary to perform it at both staging and restaging, as it allows assessment of changes in the tumor mass during treatment, as well as the presence of pathological lymph nodes [44].

The importance of restaging MRI after neoadjuvant chemoradiotherapy (CRT) has also been discussed for the management of lateral lymphadenectomy in patients who are candidates for total mesorectal excision.

Indeed, a recent study shows that a reduction of the short axis of the lymph node from 7 mm to 4 mm after neoadjuvant CRT could be a criterion for avoiding lateral lymph node dissection. In this way, lateral lymphadenectomy can be spared in about 30% of these patients. This criterion could be applied only if there is no persistent enlargement of the internal iliac lymph nodes, which would instead indicate a very high risk of recurrence [45]. Nevertheless, it has to be stressed that the highest accuracy in the differentiation of metastatic and reactive nodes is still achieved by means of other imaging techniques, in particular the FDG-PET [46].

It has also emerged that a subgroup of patients undergoing neoadjuvant chemoRT for locally advanced rectal cancer have complete (or nearly complete) tumor regression that can be visualized by MRI. Currently, it is basal MRI that defines the treatment plan, regardless of responses during treatment, so even in these good responder patients to neoadjuvant CRT, surgery is performed [47].

## Cervix

Carcinoma of the cervix is the fourth most common cancer among women worldwide [48]. External beam RT, along with concurrent chemotherapy and brachytherapy, is considered to be the standard treatment of locally advanced cervical cancer, with the first of these two techniques that have been used with different approaches, including IMRT [49]. Although currently cone beam CT is considered the most valuable procedure in clinical routine, similarly to the other X-ray-based technologies also this technique suffers from its low soft-tissue contrast compared to MRI [50]. Indeed, and especially for the pelvis (as discussed in the prostate and rectum sections) MRI is known to provide the best soft tissue discrimination within the pelvis, and has been used for repetitive imaging studies to quantify inter- and intrafraction organs and determine non-isotropic margins around the gross tumor volume and the CTV [51]. Nonetheless, it is noteworthy to mention the results of a recent study comparing CT and MRI for contouring purposes to spare functional bone marrow during IMRT therapy in patients with carcinoma cervix. In particular, it has been shown that hematological toxicity events have been observed more in the arm of subjects where images were countered using MR compared to those where a CT approach was used, with the authors concluding that MRI sequence used for bone marrow contouring might not be sufficient for sparing active bone marrow [52].

## Sarcomas

Sarcomas are a very heterogeneous group of tumors derived from mesenchymal cells, with soft tissue sarcomas (STS) representing the most common type in terms of prevalence, and that therefore will be the subject of this review [53].

The central role of MRI in the definition of pre- and post-operative RT strategies in patients with STS relies on the expected very high soft-tissue contrast resolution of this technique compared to CT, which makes this technique the gold standard for diagnostic purposes [54]. Furthermore, the occurrence of micro-metastases distant from the main localization represent also another important factor that further strengthens the diagnostic role of MRI in this condition, with these infiltrating lesions usually small in volume, but can be easily detected as areas of enhancement in T1w sequences, while the evaluation of T2w sequence allow to better depict and detect these lesions as small hyperintense areas compared to the surrounding tissues corresponding mainly to the peritumoral edema [55]. Finally, MRI is also able to detect tumor hypoxia as an early marker of RT response, allowing for. a precise dose adjustment depending on the target volume [56].

## AI-based techniques in MRI for RT planning

The integration of AI-based techniques in MRI for RT planning represents a significant advancement in the field, enhancing the precision and effectiveness of cancer treatments [57]. AI-based image acquisition methods leverage machine learning algorithms to optimize MRI parameters dynamically, leading to higher-quality images and/or reduced acquisition times. Beyond acquisition, postprocessing of MRI images using AI has also the potential to revolutionize the field. Furthermore, AI-driven algorithms can automatically segment tissues, identify tumors, and delineate organs at risk with remarkable accuracy. The availability of automated segmentation, in turn, reduces the workload on radiation oncologists and clinicians, minimizing human error and variability, while ensuring that critical structures are accurately mapped for targeted therapy. The enhanced precision in identifying tumor boundaries and sensitive structures aids in the development of more effective and individualized treatment plans [58]. Image fusion, another critical aspect of radiotherapy planning, has been greatly enhanced by AI technologies. For example, AI algorithms can merge MRI images with other imaging modalities, such as CT or PET scans, to create accurate composite images. These fused images provide a more complete understanding of the tumor environment and surrounding anatomy, facilitating precise treatment targeting. Furthermore, AI-based image-augmentation techniques have shown promising results in improving the quality of MRI images. Tools such as super-resolution imaging and noise reduction, powered by deep learning algorithms, can enhance the clarity and detail of MRI scans. This augmentation is particularly valuable in cases where high-resolution images are essential for accurate tumor delineation and treatment planning. By augmenting the images, clinicians can obtain improved visualization of the tumor and surrounding tissues, leading to more precise and effective radiotherapy treatments.

These innovations already led to higher-quality images, more accurate tumor delineation, and improved treatment precision, ultimately enhancing patient outcomes. As AI continues to evolve, its integration into MRI for RT planning is indeed expected to yield even greater advancements, further transforming the field and offering new possibilities for cancer treatment [59].

## Conclusions

In this review we provided a basic introduction for residents and non-radiologists on the physics of MR and reported evidence of the advantages and future improvements of MRI in planning a tailored radiotherapy treatment “from head to toe”, showing how MRI will increasingly become an indispensable tool not only to better characterize and evaluate lesions, but also to predict the evolution of the disease and, consequently, to ensure the best therapeutic response.

## Abbreviations

ADC: Apparent diffusion coefficient
CRT: Chemoradiotherapy
DCE: Dynamic contrast enhancement
DSC: Dynamic susceptibility contrast
DWI: Diffusion-weighted imaging
GrE: Gradient echo
IMRT: Intensity-modulated radiation therapy
LC: Lumpectomy cavity
LINACs: MRI-guided linear accelerators
M: Magnetization
MRgRT: MR-guided RT
PTV: Planning target volume
PWI: Perfusion-weighted imaging
RF: Radiofrequncy
RT: Radiation therapy
SE: Spin echo
T: Tesla
T1w: T1-weighted
T2w: T2-weighted
TE: Echo time
TR: Repetition time
